# Cognitive and behavioral benefits of newspaper reading in community-dwelling older adults: a feasibility randomized controlled trial

**DOI:** 10.3389/fragi.2026.1929793

**Published:** 2026-09-04

**Authors:** Mamoru Sato, Sawako Negoto, Shinya Nakano, Chihoko Urata, Tetsuya Ioji, Hideya Kodama, Emi Yoshimura, Yukinori Tasaki, Shinichiro Goroku, Kiichiro Morita, Yoshihisa Shoji

**Affiliations:** 1 Department of Neuropsychiatry, Kurume University School of Medicine, Kurume/Fukuoka, Japan; 2 Cognitive and Molecular Research Institute of Brain Diseases, Kurume University, Kurume/Fukuoka, Japan; 3 Biostatistics Center, Kurume University, Kurume/Fukuoka, Japan; 4 Medical Corporation Hoeikai Miyanojin Hospital, Kurume/Fukuoka, Japan; 5 Customer Service Center, The Nishinippon Shimbun Co., Ltd., Fukuoka, Japan; 6 Medical Corporation Sowakai Nakamura Hospital, Fukuoka, Japan

**Keywords:** cognitive health, dementia prevention, healthy aging, lifestyle intervention, mild cognitive impairment (MCI), newspaper reading, older adults

## Abstract

**Introduction:**

There are reports suggesting that reading newspapers is an accessible daily intellectual activity that may help prevent neurocognitive disorders, while no prospective studies have accurately investigated its effectiveness on cognitive function.

**Methods:**

This study is a randomized controlled trial designed to explore the effects of reading newspapers on cognitive function and activity levels in community-dwelling older adults. The study included 163 non-subscribers aged 60 years or older; after screening, 64 participants were selected. Participants were randomly assigned to either the newspaper reading group (n = 31) or the non-reading group (n = 32), and a 26-week randomized controlled trial (RCT) was conducted. One person was disqualified because they could not be contacted. The newspaper reading group was instructed to read the newspaper at least four days a week, and compliance was verified by checking entries on activity log sheets. In addition, both groups were assessed before and after the trial. The primary outcome was cognitive function assessed using the Japanese Version of the MONTREAL COGNITIVE ASSESSMENT (MoCA-J). Secondary outcomes included the Mini Mental State Examination (MMSE-J), the Japanese version of the Geriatric Depression Scale-15 (GDS-15-J), the Life-Space Assessment (LSA), and the Vitality Index.

**Results:**

When comparing the changes in these test items after 26 weeks, the newspaper reading group showed significantly better results than the non-reading group for the MoCA-J (difference: 1.47 [95% CI: 0.31–2.63], p = 0.013), GDS-15-J (difference: –1.85 [95% CI: –3.01 to –0.68], p = 0.002), and LSA (difference: 14.74 [95% CI: 7.13–22.35], p < 0.001). A significant trend was also observed in the newspaper reading group for the MMSE-J (difference: 2.46 [95% CI: 1.34–3.58], p = 0.065).

**Discussion:**

These findings suggest that reading newspapers is a low-cost, self-directed intervention that can be easily incorporated into preventive care for community-dwelling older adults and may have beneficial effects on cognitive function and activity levels.

## Introduction

As population aging accelerates worldwide, an estimated 57 million people were living with dementia in 2021, and approximately 10 million new cases are reported each year (World Health Organization, 2025). Japan has the highest proportion of older adults in the world. Although the Hisayama Study recently demonstrated a decline in age-adjusted dementia incidence over the past 37 years, the number of people living with dementia continues to increase because of rapid population aging. The number of people with dementia is estimated to increase from approximately 4.43 million in 2022 to 5.84 million in 2040, when one in every 6.7 older adults is projected to have dementia. Consequently, dementia prevention has become a global public health priority and the focus of extensive research (Ohara et al., 2025; Cabinet Office, 2025). The loss of cognitive function affects not only people with dementia but also the physical and mental health and well-being of caregivers and family members, making it a priority from both preventive and therapeutic perspectives (Livingston et al., 2020). Therefore, as life expectancy increases and our society continues to age, we believe it is critically important to identify factors that can help mitigate cognitive decline in older adults. Furthermore, while the development of disease-modifying drugs for dementia is progressing, there are no drugs that cure dementia itself; therefore, new strategies to prevent or delay the onset of dementia in the elderly are a critical challenge. Reports on risk factors for dementia and preventive measures have been published, including a report by an international team in The Lancet (Su et al., 2022) and the WHO’s “Guidelines on Risk Reduction for Cognitive Decline and Dementia” (World Health Organization, 2026). Based on these reports, the following measures are considered important for the prevention of dementia: 1. maintaining healthy lifestyle habits, 2. promoting social and intellectual activities, and 3. addressing mental health. Although research on preventing cognitive decline in older adults has accumulated evidence showing that improvements in physical activity and lifestyle habits are associated with the maintenance and improvement of cognitive function and a reduced risk of dementia (Song et al., 2022; Almeida-Meza et al., 2021; Northey et al., 2018; Baker et al., 2025), studies investigating participation in social and cognitive activities remain scarce and insufficient. Therefore, it is important to verify which types of social and cognitive activities are effective in preventing cognitive decline. Previous studies have reported that participation in intellectually stimulating leisure activities (such as reading) is beneficial for maintaining and improving cognitive function (Chang et al., 2021; Sörman et al., 2018; Gallucci et al., 2009; Verghese et al., 2003). However, many of these studies are observational and are susceptible to confounding factors, making them insufficient to establish a causal relationship between intellectually stimulating activities and cognitive function. On the other hand, the ACTIVE study (Tennstedt and Unverzagt, 2013), one of the most robust studies evaluating intellectual activities and cognitive function, is a multicenter randomized controlled trial (RCT) involving approximately 2,800 healthy older adults that provides strong evidence of the benefits of cognitive training on cognitive function. In this study, three types of cognitive training interventions—targeting memory, reasoning, and visual processing speed—were conducted over 5–6 weeks in 10 sessions of 60–75 min each. The study reported improvements in cognitive function in the short term and maintenance of cognitive function and IADL at 5- and 10-year follow-ups (Rebok et al., 2014; Feger et al., 2020). Furthermore, an RCT combining reading and arithmetic (Uchida and Kawashima, 2008) showed significant improvements in participants’ MMSE, Frontal Assessment Battery, and Digit Symbol Coding scores after 6 months, while an RCT (Nouchi et al., 2016) that combined reading Haiku (A short Japanese poem) and fairy tales with simple arithmetic reported beneficial effects in the intervention group on executive function, episodic memory, attention, and processing speed. Thus, while it is clear that intellectual cognitive stimulation has a positive effect on cognitive function, the issue is whether such cognitive training can be sustained. In this study, we selected newspaper reading as a form of intellectual activity that is easy to maintain on a daily basis, with the aim of clarifying its effects on changes in cognitive function and scope of activity. One reason for this is that, as of October 2025, the number of newspapers published in Japan was 0.42 copies per household; although this figure has been declining in recent years, it still amounts to approximately one copy for every 2.4 households (Pressnet, 2025). Consequently, many households have incorporated newspaper reading into their daily routines, making it readily accessible (Pressnet, 2025). Previous observational studies have shown that intellectual engagement, including newspaper reading, is associated with preserved cognitive function in older adults (Chang et al., 2021; Verghese et al., 2003; Hughes et al., 2010; Sugita et al., 2021; Wilson et al., 2002). Previous observational studies have shown that intellectual engagement, including newspaper reading, is associated with preserved cognitive function in older adults. Newspaper reading is a low-cost, lifestyle-integrated, self-directed, non-pharmacological activity that is readily accessible to community-dwelling older adults and has the potential to serve as a scalable cognitive intervention. Its low cost, accessibility, and ease of integration into daily life make it a promising intervention with potential applications in public health and dementia prevention. Therefore, we conducted a randomized controlled trial to investigate the effects of newspaper reading on cognitive function and activity levels in community-dwelling older adults.

## Materials and methods

### Subject and study design

To examine changes in cognitive function and indicators of motivation and activity resulting from newspaper reading among community-dwelling older adults aged 60 and older, this study enrolled participants with mild cognitive impairment who had not read newspapers in the past 6 months, had no history of conditions such as cerebral infarction or hemorrhage, and had not been diagnosed with dementia or depression. We conducted a 26-week randomized controlled trial (RCT). The study was conducted from May 2024 to August 2025. To promote adherence throughout the intervention and minimize attrition, participants in the newspaper-reading group were asked to submit monthly activity logs documenting their newspaper-reading frequency. These logs were reviewed monthly to confirm continued participation in the intervention. Participants in the control group were contacted by telephone once a month to confirm the absence of major changes in their health status. All monthly follow-up procedures were conducted by research assistants who were not directly involved in the study assessments or intervention delivery.

### Participant recruitment and location

Recruitment of study participants began in February 2024. Participants were recruited through advertisements, community outreach activities conducted by the research team at facilities such as paid nursing homes, day care centers, senior citizens’ clubs, and dementia cafés, and the distribution of approximately 3,100 flyers to local residents. All participants were recruited from within Kurume City. For this study, screening tests for study enrollment were conducted in two stages (primary and secondary). The primary screening tests were held at four locations—Kurume University Hospital, the Kurume City Civic Activity Support Center, Kurume City Hall, and a room in a local shopping mall—to accommodate the participants’ schedules and locations. The secondary screening tests were all conducted at Kurume University Hospital.

### Screening tests and eligibility

Participants underwent two screening tests prior to enrollment. In the initial screening, the Mini Mental State Examination—Japanese version (MMSE-J) (Sugishita et al., 2018) and the Japanese version of the Geriatric Depression Scale-15 (GDS-15-J) (Sugishita et al., 2017) were administered to assess cognitive function and depression or depressive symptoms. Based on the results of these tests, participants with MMSE scores ranging from 24 to 27 and GDS-15-J scores below 10 proceeded to the second screening. At the same time, a participant questionnaire was administered to gather information on newspaper reading history, lifestyle history (years of education, work history, exercise habits, smoking, and alcohol consumption), and the presence of physical comorbidities (hypertension, dyslipidemia, diabetes, respiratory diseases, cerebrovascular disorders, heart disease, kidney disease, etc.). In the secondary examination, a general assessment was conducted using the Japanese Version of the MONTREAL COGNITIVE ASSESSMENT (MoCA-J) (Fujiwara et al., 2010), the Life-Space Assessment (LSA) (Baker et al., 2003) from the Elderly Status Assessment Set (E-SAS), and the Vitality Index (Toba et al., 2002). In addition, during the secondary examination, head CT scans were performed on all subjects to thoroughly examine intracranial lesions that could potentially affect cognitive impairment. All tests were conducted by trained clinical psychologists and psychiatrists employed at our hospital. Exclusion criteria included as following, being under 60 years of age; inability to provide written informed consent; having a newspaper subscription within the past 6 months; visual impairment making reading difficult; MMSE-J score of 23 or below or 28 or above; currently receiving treatment for a diagnosed dementia; abnormal findings on a head CT scan; GDS-15-J score of 10 or higher indicating suspected depression, or the presence of other mental disorders or substance abuse (including past history). During the secondary screening, all participants underwent a clinical evaluation by an experienced psychiatrist. Cognitive status was determined according to the Petersen criteria (Petersen, 2004), and participants were classified as having mild cognitive impairment (MCI), dementia, or normal cognition. Only participants with MCI were included in the study, while those with dementia or normal cognition were excluded. Participants who met the inclusion criteria for this study and received medical approval for participation from the principal investigator were considered eligible and were randomly assigned to either the newspaper reading group (intervention group) or the group continuing their usual daily lives (control group). Signed informed consent regarding this study was obtained from all participants. This study was conducted with the approval of the Ethics Committee of Kurume University Hospital (Study No.: 24083). All participant names were blinded using participant numbers by staff not involved in the examinations or evaluations.

### Randomization

Allocation concealment was ensured using sequentially numbered, opaque, sealed envelopes (SNOSE) prepared by an independent researcher not involved in participant recruitment. Each envelope was prepared in advance, contained a single allocation card, and was sealed with adhesive and light-blocking foil. The envelopes were opened sequentially after obtaining participant consent and confirming registration. The envelopes were stored under lock and key, and the date, time, and name of the person opening them were recorded in a dedicated logbook. The generation and implementation of the allocation sequence were performed by personnel independent of those responsible for participant registration (in accordance with CONSORT Item 18) (Schulz et al., 2010).

### Outcomes and assessment measures

The primary outcome was an assessment of cognitive function using the MoCA-J after 26 weeks of newspaper reading. Similarly, secondary outcomes were assessed using the MMSE, GDS-15-J, LSA, and Vitality Index at 26 weeks. The MoCA-J and MMSE-J were used to assess cognitive function. The MoCA-J is the Japanese version of the MoCA (Nasreddine et al., 2005), an international screening tool developed in 2005 to detect cognitive decline, including mild cognitive impairment, with high sensitivity. Because it assesses not only memory impairment but also executive function, attention, and abstract thinking, it is particularly effective at detecting early changes that are often missed by the MMSE. The MMSE-J is the Japanese version of the MMSE (Folstein et al., 1975), which is widely used internationally in clinical settings as an initial screening test for cognitive decline, and it has good reliability and validity. The GDS-15-J was used to differentiate depressive states. This is the Japanese version of the GDS (Yesavage et al., 1982), which was developed as a screening scale specifically for late-life depression. It is less susceptible to the influence of physical symptoms and features a two-choice response format that is easy for older adults to understand. Typically, scores of 0–4 are considered normal, 5–9 indicate a depressive tendency, and 10 or higher suggest suspected depression.

For the assessment of activity range, the LSA subscale from the E-SAS was used. The E-SAS is a comprehensive assessment battery designed to provide a multifaceted and convenient evaluation of the physical, mental, and daily living functions of older adults; it is primarily used in long-term care and medical settings. The LSA quantifies the spatial range an individual actually moves within over a given period, taking into account frequency and level of independence (presence or absence of assistive devices or personal assistance), and rates this on a scale of 0–120 points.

The Vitality Index was used to assess motivation. The Vitality Index is a scale primarily designed to conveniently assess the motivation and spontaneity of older adults. It evaluates five items—getting out of bed, communication, eating, toileting, and daytime activities—on a scale of 0–2 points each, with a total score ranging from 0 to 10 points; a higher score indicates greater vitality.

### Intervention methods

The newspaper reading group received free morning editions of the Nishinippon Shimbun for 26 weeks. For the purposes of this study, newspaper reading was operationally defined as reading newspaper articles on at least 4 days per week during the 26-week intervention period. Participants were instructed to read at least one article of interest from the front page, read the front-page column aloud, and complete the newspaper quiz. To verify whether they had actually read the articles, participants were asked to write down the main points of the content on a reading log sheet, which was then reviewed by an evaluator. In the control group, regular newspaper reading was prohibited during the study period, and participants were instructed to otherwise continue their normal daily lives. In this study, there was no social contact whatsoever between the study staff and participants during the study period. At the end of the 26-week trial period, all participants underwent the same tests at Kurume University Hospital that had been administered before the trial began (MoCA-J, MMSE-J, GDS-15-J, LSA, Vitality Index).

### Statistical analysis

Baseline demographic characteristics between the intervention and control groups were compared using the chi-square test for categorical variables and Welch’s t-test for continuous variables. Changes in test results before and after the intervention were compared using Welch’s t-test. Although this study is a randomized trial and confounding factors are theoretically controlled, sensitivity analyses adjusted for covariates were performed to demonstrate the robustness of the primary analysis. In this study, sensitivity analyses were conducted using gender, age, and years of education—variables that could influence the response variable—as explanatory variables. The statistical significance level was set at less than 5%. All statistical analyses were performed using JMP Pro ver. 16 (SAS Institute Inc., Cary, NC, USA).

To clarify the hypothesized causal relationships among the intervention, outcomes, potential mediators, and covariates, a directed acyclic graph (DAG) was developed (Supplementary Figure S1). Because participants were randomly allocated to the intervention and control groups, baseline covariates were, in principle, independent of the exposure. Nevertheless, age, sex, and years of education were included as covariates in the adjusted sensitivity analyses based on the hypothesized causal structure and previous evidence to improve the precision of the estimated intervention effect by accounting for potential chance imbalances between groups (ICH E9) (International Council for Harmonisation of Technical Requirements for Pharmaceuticals for Human Use ICH, 1998). Potential mediators were not included in the adjusted models to avoid overadjustment bias.

## Results

### Participant profile

**FIGURE 1:**
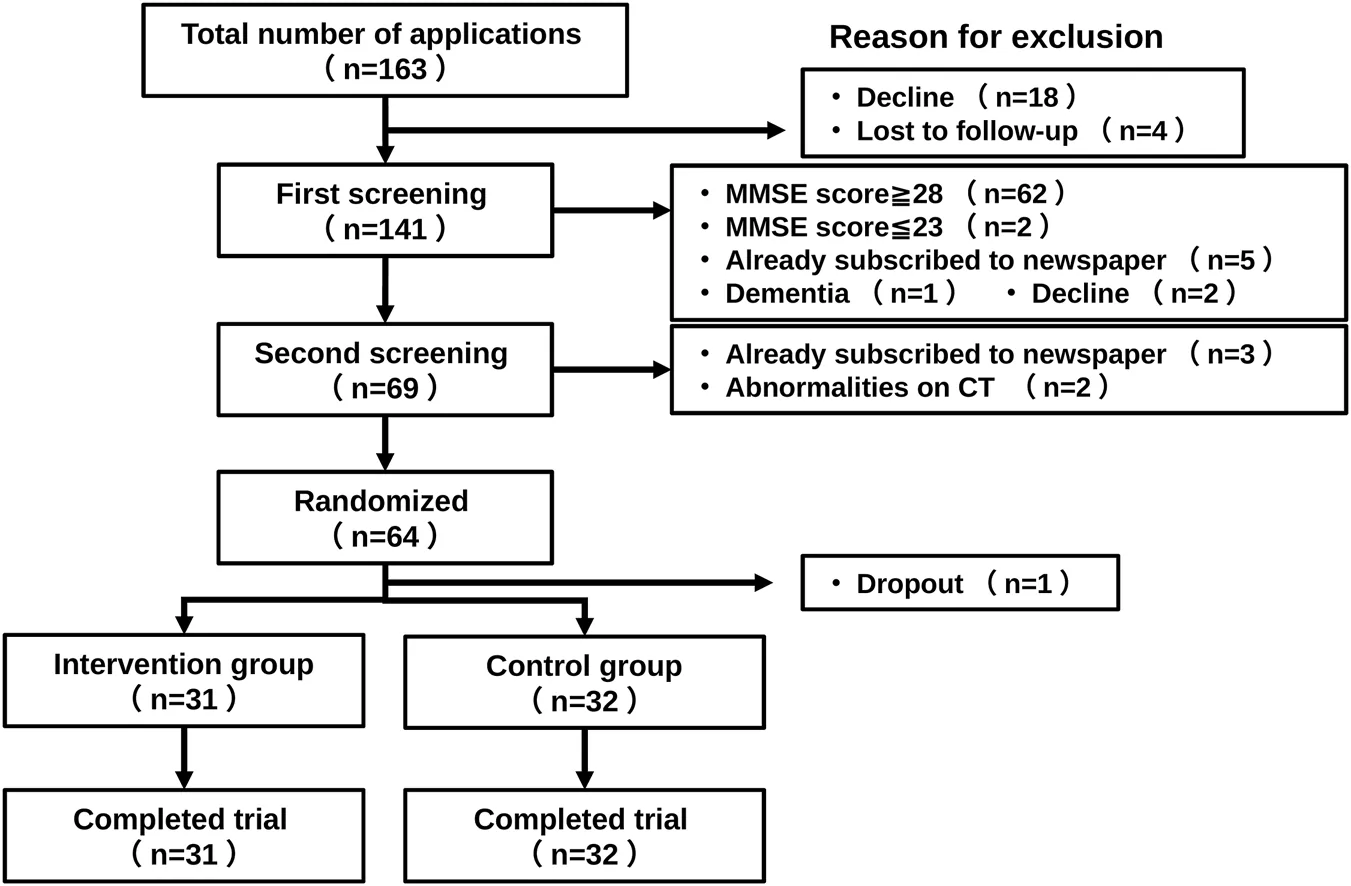
CONSORT flow diagram. This figure shows the number of participants who underwent eligibility assessment, the number of participants randomized, the number of participants assigned to each study group, the number of participants who dropped out of the study, and the number of participants included in the final analysis.

A total of 163 individuals applied for this study. By the time of the initial screening, 22 participants had been excluded due to withdrawal or loss of contact. Of the 141 participants who underwent the initial screening, 72 were excluded (62 scored 28 or higher on the MMSE, 2 scored 23 or lower, 5 were already newspaper subscribers, 1 had dementia, and 2 could not schedule the secondary screening). Of the 69 participants who proceeded to the second screening, 5 were excluded (3 were found to have read newspapers since the first screening, and 2 had abnormal findings on head CT scans). The abnormalities detected on head CT scans were a meningioma and a chronic subdural hematoma; the patients were referred to neurosurgery, respectively. Ultimately, 64 participants were deemed eligible and randomly assigned to one of two groups: the newspaper reading group (intervention group) with 31 participants and the non-reading group (control group) with 33 participants. One participant in the non-reading group subsequently lost contact, resulting in a final group size of 32. All randomized participants were living independently in the community. Figure 1 shows the CONSORT diagram for the participants throughout the study. A total of 63 subjects completed this study; by gender, there were 45 women (71.4%), The mean age, MMSE-J, MoCA-J, and GDS-15-J scores were 73.3 ± 7.6 years, 26.8 ± 1.3, 24.8 ± 3.1, and 2.7 ± 2.2, respectively. Table 1 shows the baseline characteristics of the intervention group (N = 31) and the control group (N = 32). No significant differences were observed between the two groups in terms of gender, age, educational level, habitual occupation, physical activity, alcohol consumption, smoking, physical illness, MMSE-J, MoCA-J, GDS-15-J, LSA, or Vitality Index scores.

**TABLE 1:** Baseline characteristics.

Characteristics	Intervention group (n = 31)	Control group (n = 32)	p-value
Sex (female), n (%)	23 (74.1%)	22 (68.7%)	0.63
Age (years), (SD)	73.1 ± 7.5	73.5 ± 7.7	0.81
Education (years), (SD)	13.1 ± 1.9	13.3 ± 1.9	0.76
Work, n (%)	7 (22.6%)	9 (28.1%)	0.49
Exercise, n (%)	14 (45.2%)	12 (37.6%)	0.54
Alcohol, n (%)	11 (35.5%)	6 (18.7%)	0.14
Smoking, n (%)	3 (9.77%)	4 (12.6%)	0.73
Physical complications			
Hypertension, n (%)	10 (32.3%)	15 (46.8%)	0.24
Diabetes, n (%)	1 (3.23%)	6 (18.7%)	0.05
Dyslipidemia, n (%)	8 (26%)	7 (21.8%)	0.72
Respiratory disease, n (%)	0	0	N/A
Cerebrovascular disease, n (%)	1 (3.23%)	2 (6.25%)	0.58
Cardiovascular disease, n (%)	5 (16.1%)	7 (21.8%)	0.57
Kidney disease, n (%)	0	0	N/A
Average test results			
MMSE-J score, (SD)	26.7 ± 1.21	27.0 ± 1.3	0.48
GDS-15-J score, (SD)	2.96 ± 2.07	2.34 ± 2.2	0.26
MoCA-J score, (SD)	25.0 ± 2.76	24.6 ± 3.1	0.62
LSA score, (SD)	85.3 ± 19.2	84.9 ± 19.6	0.93
Vitality index score	10	10	N/A

The chi-squared test was used for sex differences, and Welch’s t-test was used to compare other variables between the two groups. Values are presented as mean ± standard deviation or number (%), as appropriate.

MMSE-J, The Japanese version of the Mini-Mental State Examination; MoCA-J, the japanese version of the montreal cognitive assessment; GDS-15-J, the japanese version of the geriatric depression scale; LSA, Life-Space Assessment.

### Analysis of intervention effects

Table 2 compares the changes from baseline in test results for both groups following the 26-week intervention.

**TABLE 2:** Comparison of changes from baseline to post-intervention between the intervention and control groups.

Outcome	Score (mean ± SD)		p-value
	Intervention group	Control group	
MoCA-J	0.97 ± 2.42	−0.5 ± 2.14	**0.013**
MMSE-J	1.52 ± 2.13	0.66 ± 1.41	0.065
GDS-15-J	−0.97 ± 2.38	0.88 ± 2.08	**0.002**
LSA	6.63 ± 14.7	−8.11 ± 15.5	**< 0.001**
Vitality index	−0.06 ± 0.25	−0.25 ± 0.57	0.099

Values represent changes from baseline to the post-intervention assessment and are presented as mean ± SD., Between-group differences in change scores were analyzed using Welch’s t-test. Positive values indicate improvement in MoCA-J, MMSE-J, LSA, and Vitality Index scores, whereas negative values indicate improvement in GDS-15-J scores. Statistically significant differences are shown in bold (p < 0.05).

MMSE-J, The Japanese version of the Mini-Mental State Examination; MoCA-J, the japanese version of the montreal cognitive assessment; GDS-15-J, the japanese version of the geriatric depression scale; LSA, Life-Space Assessment.

### Cognitive outcomes

**FIGURE 2:**
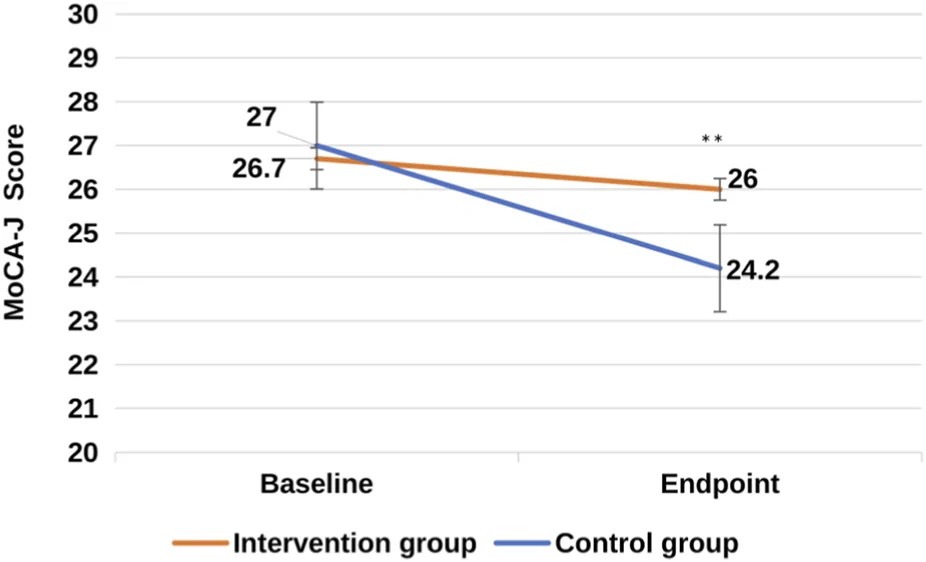
Comparison of mean MoCA-J scores at baseline and postintervention (mean ± SE). Values are presented as mean ± SE. **p < 0.01 for the between-group difference in change scores from baseline to post-intervention. MoCA-J: Japanese version of the Montreal Cognitive Assessment.

The mean change in MoCA-J scores was 0.97 (95% CI: 4.21–6.15) in the intervention group, while in the control group it was −0.5 (95% CI: 4.87 – 3.87). The intervention group showed better results than the control group on the MoCA-J (difference: 1.47 [95% CI: 0.31–2.63], p = 0.013). Figure 2 shows the changes in MoCA-J scores from baseline to post-intervention. As the prespecified primary outcome, MoCA-J was selected for graphical presentation, whereas the remaining secondary outcomes are presented in Table 2. Regarding the mean change in MMSE-J scores, the intervention group showed changes of 1.52 (95% CI: 2.84–5.85) and 0.66 (95% CI: 0.15–1.17). Although the intervention group tended to show better results than the control group, no statistically significant difference was observed.

### Psychological outcomes

The GDS-15-J score in the intervention group was −0.97 (95% CI: 5.82 to 3.88), whereas that in the control group was 0.88 (95% CI: 0.33–5.09). In terms of outcome measures, the intervention group showed significantly better results than the control group (difference in GDS-15-J: 1.85 [95% CI: 3.01 to −0.68], p = 0.003).

### Functional outcomes

In the LSA analysis, the score for the intervention group was 6.63 (95% confidence interval: 23.30–36.56), whereas the score for the control group was −8.11 (95% confidence interval: 39.73–23.51). For these outcome measures, the intervention group showed significantly better results than the control group (difference in LSA: 14.74 [95% CI: 7.13–22.35], p < 0.001). Regarding the Vitality Index, the change in the intervention group was −0.06 (95% confidence interval: −0.57 to 0.45), and the change in the control group was −0.25 (95% confidence interval: −1.41 to 0.91). Although the intervention group showed a tendency toward better results than the control group, no statistically significant difference was observed.

The results of the sensitivity analysis are presented in the supplemental data.

## Discussion

**FIGURE 3:**
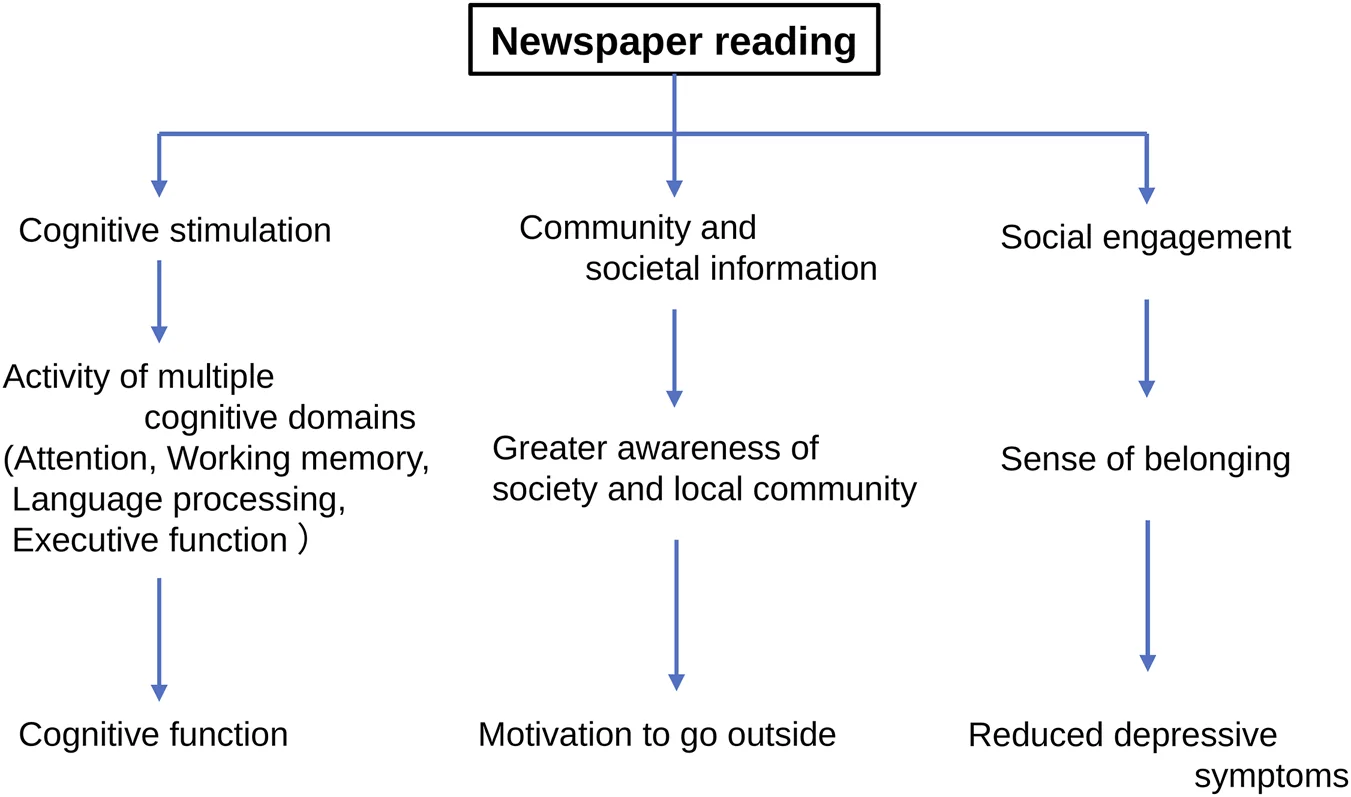
Conceptual framework illustrating the potential pathways through which newspaper reading may promote healthy aging in community-dwelling older adults. Newspaper reading may provide cognitive stimulation while increasing social engagement and access to current and community information. These pathways may contribute to reduced depressive symptoms and expanded life-space mobility, thereby supporting the maintenance of cognitive function. The proposed pathways are hypothetical and are intended to provide a conceptual framework for interpreting the findings of the present study.

The purpose of this study was to investigate whether habitual newspaper reading contributes to the maintenance or improvement of cognitive function in older adults living in the community. While previous reports have suggested that newspaper reading plays a protective role in cognitive function, prior research had several limitations. First, many studies grouped newspaper reading together with general reading within the same category of cognitive activities (Sörman et al., 2018; Sugita et al., 2021; Wilson et al., 2002; Scarmeas et al., 2001). Since the differences in the effects on cognitive function between reading books and reading newspapers—both of which involve the act of reading—have not been investigated, we believe that research focused specifically on newspaper reading is important. Generally, reading books requires maintaining a continuous flow of information while continuously understanding the story’s content, whereas newspapers are fragmented and cover a wide range of topics; therefore, they are thought to require greater shifts in attention and memory, and the cognitive domains involved may also differ. For this reason, this study focused specifically on newspaper reading within the broader act of reading. Furthermore, in previous studies, whether participants engaged in newspaper reading was determined through questionnaires (or evaluator interviews), and the actual act of reading was not verified. Therefore, in this study’s design, to ensure that participants read newspapers at least 4 days a week, we had them write down the main points of the content they read, and evaluators verified this to confirm the evidence of reading. In this study, participants were recruited primarily based on their baseline cognitive function levels, focusing on those with mild cognitive impairment. The rationale for this was to avoid the ceiling effect—a phenomenon in which, when healthy older adults are studied, their scores on cognitive function tests have already reached the upper limit, leaving little room for improvement even with intervention and making it difficult to observe significant changes. Furthermore, at the level of mild cognitive impairment, while neurodegeneration has already begun, synaptic plasticity is partially preserved, suggesting the potential for intervention effects to be reflected in the short term (Di Lorenzo et al., 2020; Li and Selkoe, 2020). Indeed, compared to the control group, a greater number of participants in the intervention group showed an increase or maintenance of their MoCA-J scores. Although no significant difference was observed between the two groups on the MMSE-J, a significant trend was observed. Furthermore, this study utilized several assessment scales to demonstrate secondary effects beyond cognitive function. First, depressive symptoms were assessed using the GDS-15-J. The purpose of this was to investigate whether reading newspapers would yield similar results, given the numerous previous studies indicating that leisure activities have a beneficial effect on depressive symptoms (Takeda et al., 2015; Fancourt et al., 2020; Wang et al., 2024). In a longitudinal study examining the association between leisure activities and depressive symptoms, Bone et al. demonstrated that specific activities such as baking, cooking, and participation in clubs or projects reduce the risk of developing depression, while reporting that reading was not associated with this risk (Bone et al., 2022). On the other hand, while this study found a significant improvement in the GDS-15-J score in the intervention group, this may largely be due to the difference between reading and the medium of newspapers. Reading a newspaper is not merely an act of reading but involves continuous engagement with current events and social information unfolding in the real world. Furthermore, it is thought to contribute to maintaining interest in and a sense of belonging to the local community; compared to general reading, it can be described as an activity that enhances connectivity to the real world, and it is conceivable that it increases the reader’s psychological satisfaction. Indeed, there are reports indicating that cultural activities involving media consumption, including newspapers, yield positive outcomes regarding anxiety, depression, and life satisfaction (Lee et al., 2012; Cuypers et al., 2012). In line with this, the present study found a significant trend toward improvement in the LSA score through newspaper reading, indicating that the scope of daily activities among older adults had expanded. It was suggested that newspaper reading may have a positive effect on the expansion of living space (LSA) by increasing exposure to local information and social interest, thereby increasing the purpose and frequency of going out. This may also have a potential correlation with the aforementioned improvement in depression. The hypothesized mechanisms underlying these findings are summarized in Figure 3. Although these mechanisms were not directly examined in the present study, they provide a conceptual framework for interpreting the observed findings and should be investigated in future studies. On the other hand, no significant difference in Vitality Index scores was observed between the two groups. One possible reason for this is that the baseline Vitality Index scores in both groups were high, which may have led to a ceiling effect. Additionally, since the Vitality Index primarily assesses areas related to ADLs such as getting out of bed, eating, and toileting, it was considered difficult to elicit changes in a group that was already independent over a limited period of 26 weeks. The results of this study remained significant even after conducting sensitivity analyses adjusted for gender, age, and years of education. These factors were included as adjustment variables because previous studies have shown that women are more likely to engage in social and cognitive leisure activities (Finkel et al., 2018), age is one of the most powerful risk factors for dementia (Prince et al., 2013; Breijyeh and Karaman, 2020; Fang et al., 2025), and there is substantial evidence supporting the notion that low educational attainment increases the risk of dementia, while high educational attainment builds cognitive reserve and contributes to a reduced risk of dementia onset (Sharp and Gatz, 2011; Stern, 2012; Walters et al., 2025). This study had several limitations. First, the study period was only 6 months; while the preventive effects on cognitive decline were demonstrated over this short period, it remains unclear how long these effects will last. Therefore, we believe that longitudinal studies spanning several years are necessary in the future. Second, there is no definitive evidence regarding the frequency at which habitual intellectual activities must be performed to yield benefits. For example, some studies use broad scales for reading habits ranging from “occasionally” to “daily” per week (Sörman et al., 2018), while others report that even reading once a week can prevent cognitive decline (Chang et al., 2021). Given this, the reading habit of four or more days per week observed in this study may have a particularly strong effect on preventing cognitive decline. Third, the study population consisted predominantly of women, which may limit the generalizability of our findings. As discussed above, women are generally more likely than men to engage in social and cognitively stimulating leisure activities (Finkel et al., 2018), which may have contributed to the higher proportion of female participants in this study. However, because the present study was not designed to evaluate sex-specific differences in the effects of newspaper reading, it remains unclear whether the observed benefits are equally applicable to both men and women. Future studies involving larger, more sex-balanced and diverse populations are needed to confirm the generalizability of these findings. Fourth, the observed outcomes may have been influenced by the specific assessment instruments used in this study. Different instruments may vary in their sensitivity to changes in cognitive function, depressive symptoms, activity range, and motivation, and therefore may yield different findings. Future studies using alternative validated measures are needed to confirm the robustness and generalizability of our findings. Finally, there is insufficient evidence regarding the age range for which newspaper reading is beneficial. Studies of healthy older adults have reported that reading comprehension declines due to age-related cognitive decline (attention, working memory, processing speed) (Pegoraro et al., 2024), with this trend becoming particularly pronounced in those aged 80 and older (Riffo et al., 2024). The participants in this study spanned a wide age range from 62 to 90 years. Although we performed age-adjusted sensitivity analyses, we believe that conducting the intervention in groups stratified by baseline age would have allowed for a more detailed clarification of the benefits of newspaper reading. Due to limitations in sample size, we were unable to fully conduct subgroup analyses or examine interactions, and caution is required in interpreting the results. Therefore, verification through even larger-scale studies in the future is desirable. Despite the limitations mentioned above, our study yielded many positive findings. It suggests that reading newspapers is an effective lifestyle intervention for maintaining cognitive function, preventing depression, and expanding the scope of activities among older adults; we believe these findings should be verified in future studies with larger sample sizes and adequate statistical power. While our study offers promising preliminary findings, it is also clear that many other important factors exist for older adults to maintain cognitive function, including regular exercise, prevention of lifestyle-related diseases, prevention of mental disorders such as depression, social interaction, and various leisure activities. Reading newspapers is merely one form of cognitive activity that provides cognitive stimulation, and we believe its protective effects on cognitive function will be further strengthened when combined with other protective factors.

## Data Availability

The raw data supporting this article has not been made publicly available for ethical and privacy reasons.

## Supplementary material

SUPPLEMENTARY FIGURE S1 Hypothesized causal structure linking newspaper reading and cognitive function: A directed acyclic graph (DAG). Directed acyclic graph (DAG) illustrating the hypothesized causal relationships among the newspaper-reading intervention, cognitive outcomes, potential mediators, and baseline covariates. Cognitive stimulation, cognitive reserve, social engagement, and access to current and community information were considered potential mediators and were not adjusted for in the analyses to avoid overadjustment bias. U denotes unmeasured confounders.
