# Correction: Cognitive and behavioral benefits of newspaper reading in community‐dwelling older adults: a feasibility randomized controlled trial

**DOI:** 10.3389/fragi.2026.1992402

**Published:** 2026-09-21

**Authors:** Mamoru Sato, Sawako Negoto, Shinya Nakano, Chihoko Urata, Tetsuya Ioji, Hideya Kodama, Emi Yoshimura, Yukinori Tasaki, Shinichiro Goroku, Kiichiro Morita, Yoshihisa Shoji

**Affiliations:** 1 Department of Neuropsychiatry, Kurume University School of Medicine, Kurume/Fukuoka, Japan; 2 Cognitive and Molecular Research Institute of Brain Diseases, Kurume University, Kurume/Fukuoka, Japan; 3 Biostatistics Center, Kurume University, Kurume/Fukuoka, Japan; 4 Medical Corporation Hoeikai Miyanojin Hospital, Kurume/Fukuoka, Japan; 5 Customer Service Center, The Nishinippon Shimbun Co., Ltd., Fukuoka, Japan; 6 Medical Corporation Sowakai Nakamura Hospital, Fukuoka, Japan

**Keywords:** cognitive health, dementia prevention, healthy aging, lifestyle intervention, mild cognitive impairment (MCI), newspaper reading, older adults

There was a mistake in Figure 2 as published. The position of the asterisk indicating statistical significance was incorrect. The corrected Figure 2 appears below.

**FIGURE 2:**
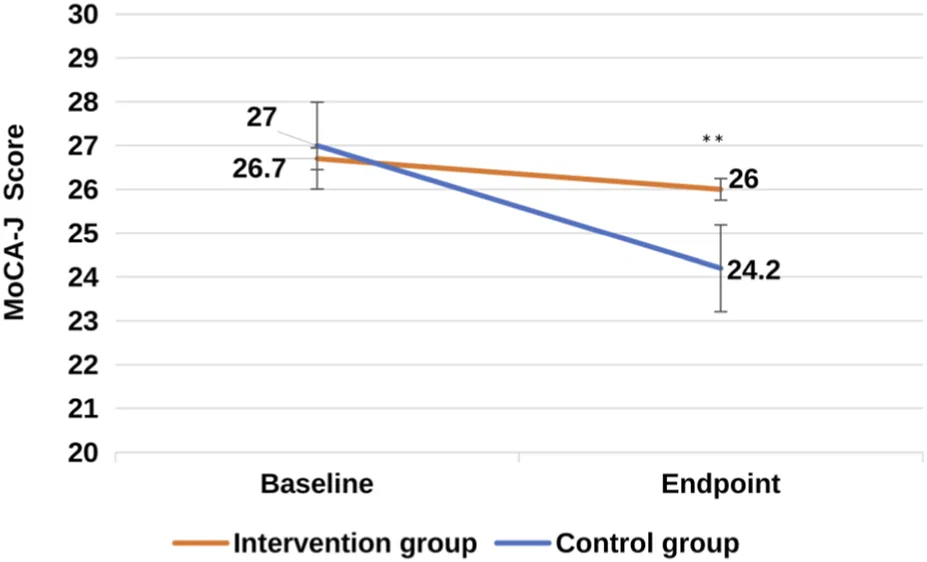
Comparison of mean MoCA-J scores at baseline and postintervention (mean ± SE). Values are presented as mean ± SE. **p < 0.01 for the between-group difference in change scores from baseline to post-intervention. MoCA-J: Japanese version of the Montreal Cognitive Assessment.

The original version of this article has been updated.

